# Tuning transcription factor DegU for developing extracellular protease overproducer in *Bacillus pumilus*

**DOI:** 10.1186/s12934-023-02177-0

**Published:** 2023-08-27

**Authors:** Chao-Ying Xie, Wen-Jin Li, Hong Feng

**Affiliations:** Key Laboratory for Bio-resources and Eco-Environment of the Ministry of Education, Sichuan Key Laboratory of Molecular Biology and Biotechnology, College of Life Sciences, Sichuan University, Chengdu, 610064 People’s Republic of China

**Keywords:** *B. pumilus*, DegU, Global transcription machinery engineering, Alkaline protease

## Abstract

**Background:**

Global transcription machinery engineering (gTME) is an effective approach employed in strain engineering to rewire gene expression and reshape cellular metabolic fluxes at the transcriptional level.

**Results:**

In this study, we utilized gTME to engineer the positive transcription factor, DegU, in the regulation network of major alkaline protease, AprE, in *Bacillus pumilus*. To validate its functionality when incorporated into the chromosome, we performed several experiments. First, three negative transcription factors, SinR, Hpr, and AbrB, were deleted to promote AprE synthesis. Second, several hyper-active DegU mutants, designated as DegU(hy), were selected using the fluorescence colorimetric method with the host of the *Bacillus subtilis* Δ*degSU* mutant. Third, we integrated a screened *degU*(L113F) sequence into the chromosome of the Δ*hpr* mutant of *B. pumilus* SCU11 to replace the original *degU* gene using a CRISPR/Cas9 system. Finally, based on transcriptomic and molecular dynamic analysis, we interpreted the possible mechanism of high-yielding and found that the strain produced alkaline proteases 2.7 times higher than that of the control strain (*B. pumilus* SCU11) in LB medium.

**Conclusion:**

Our findings serve as a proof-of-concept that tuning the global regulator is feasible and crucial for improving the production performance of *B. pumilus*. Additionally, our study established a paradigm for gene function research in strains that are difficult to handle.

**Supplementary Information:**

The online version contains supplementary material available at 10.1186/s12934-023-02177-0.

## Background

Alkaline proteases are of the important enzymes in several industries, including food, detergent, feed, waste, and leather [1]. It accounts for more than 60% of total industrial enzyme sales [2]. As the market demands higher yield, efficient alkaline protease-producing strains with resistance to high temperature and alkalinity (“four-high”) have become the focus of recent research [3, 4].

The genus *Bacillus* is home to many efficient alkaline protease producers [5], with *B. subtilis* emerging as an attractive host for expression due to its desirable properties [6]. Several studies have shown that the regulation of alkaline protease (AprE) in *B. subtilis* 168 primarily occurs at transcriptional level by various activators and repressors directly, such as DegU ~ Pi, SinR, ScoC (also known as Hpr), AbrB, among others [7–10]. In addition, it was indirectly regulated by the other proteins, including Spo0A ~ Pi, AbbA (an inhibitor of AbrB), phosphorylated SalA and TnrA, DegQ (an activator of DegU phosphorylation), DegR (a protector of DegU ~ Pi), RapG (an inhibitor of DegU ~ Pi), as well as by factors that control the activities of the indirect regulators, such as the phosphor-relay components, the kinase for SalA, glutamine synthetase (an inhibitor of TnrA), and PhG [11–16].

The DegSU system of *B. subtilis* comprises two components, the membrane-associated histidine kinase, DegS, and the cytoplasmic response regulator, DegU [17–20]. The former detects the signal or stress, while the latter controls cellular response including genetic competence [17], activation and inhibition of motility [21], activation and inhibition of biofilm formation, etc. [22, 23]. DegU was the crucial transcriptional activator of *aprE*, and its deficiency or hyper-secretion type leads to deficient or excess production of degradative enzymes [24]. The classical mutation type, *sacU*32(Hy)with a H12L mutation in DegU was applied to promote several secreted enzyme biosynthesis [25–28]. In 2004, key residues for DegU binding to *aprE* promoter was probed by alanine-scanning analysis [29].

As a tool for modifying microbial metabolic pathways, transcription factors have the unique advantage of “multi-point regulation,“ which compensates for the insufficient effect of a single gene modification in metabolic engineering operations [30]. Global transcription machinery engineering (gTME) is a method for modifying transcription factors related to metabolic pathways, triggering gene network and cellular metabolic network reprogramming, altering transcriptional efficiency, and resulting in overall change of gene expression at the transcriptional level [31, 32]. gTME has attracted significant attention in recent years due to its efficient application in altering gene transcription to obtain beneficial cellular phenotypes [33, 34].

**Fig. 1:**
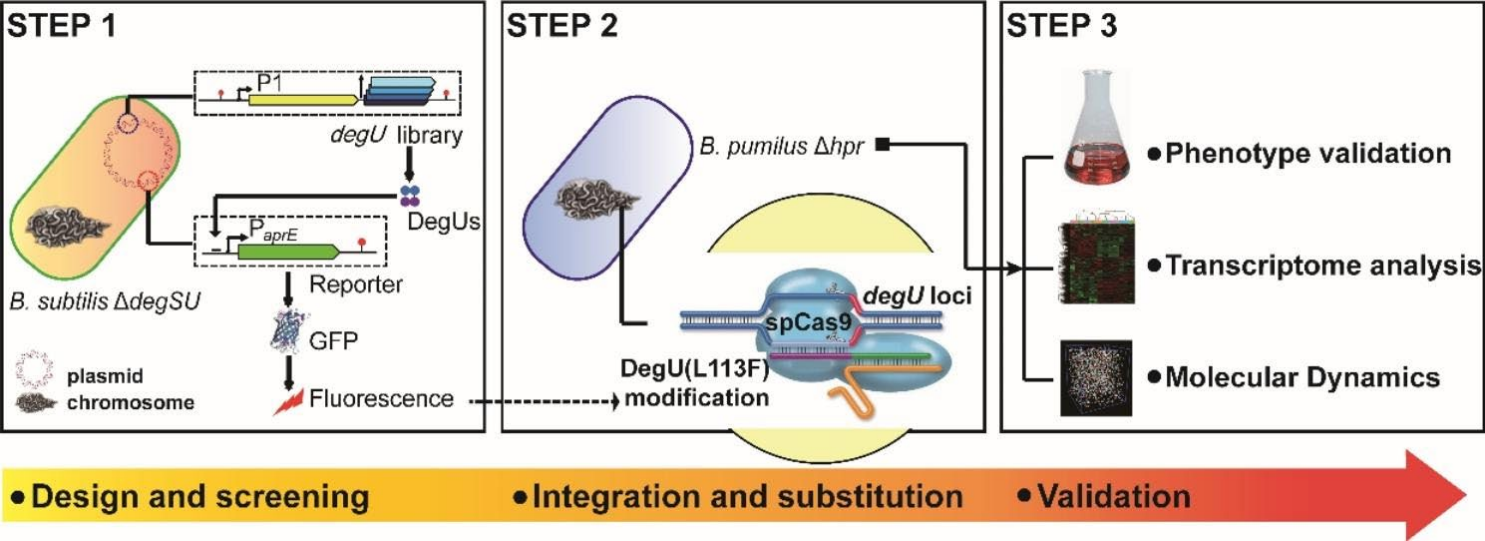
Schematic diagram of the whole process for elevating the extracellular protease activity in *B. pumilus*

*B. pumilus* is considered an excellent cell factory [3, 35]. *B. pumilus* BA06 was isolated from daily waste in our laboratory and it produced an extracellular alkaline protease with dehairing function during leather processing [36], which is encoded by an ortholog gene of the *aprE* gene in *B. subtilis* and accounts for over 70% extracellular alkaline protease activity in *B. pumilus* [37]. However, knowledge of transcriptional regulation of *aprE* in *B. pumilus* was in its infant stage [38]. Therefore, we adopted a multi-step metabolic engineering strategy to overcome potential bottlenecks associated with AprE production (Fig. 1).

In this study, we aimed to enhance AprE production in *B. pumilus* by simultaneously engineering the positive transcription factor DegU and disrupting the negative transcription factor, Hpr. Fed-batch fermentation of the resulting high-yielding strain demonstrated both the feasibility and effectiveness of such engineering strategy and the potential application of factor DegU(L113F).

## Results

### Effect of transition factors on AprE biosynthesis

**Fig. 2:**
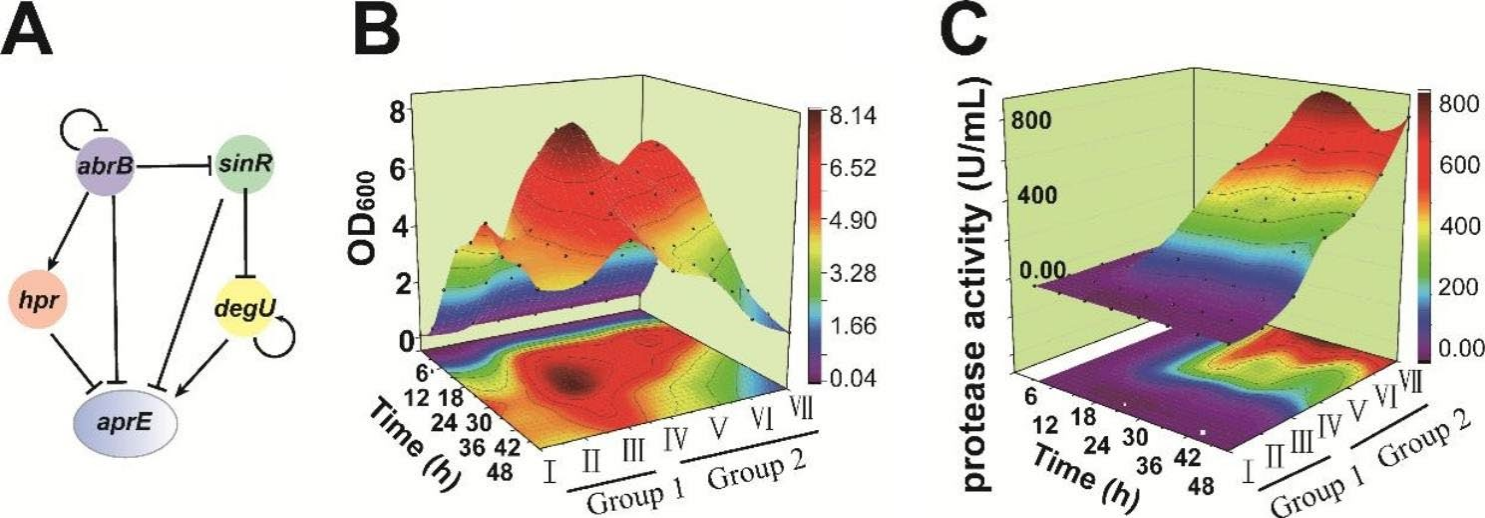
Effects of several global transcription factors on growth and extracellular alkaline protease synthesis in *B. pumilus.***(A)** The transcriptional network of *aprE* of *B. subtilis* 168 (retrieved from the subtiwiki database) [41]. **(B)** Cell growth waterfall diagram of different *B. pumilus* strains in LB medium (37 °C, 200 rpm, aerobically). **(C)** The extracellular alkaline protease activity of different *B. pumilus* strains across time. The tested bacterial strains comprise of Group 1 (including I, BA06 Δ*degSU*; II, BA06 Δ*degS*; III, BA06) and Group 2 (including IV, SCU11; V, SCU11 Δ*sinR*; VI, SCU11 Δ*hpr*; VII, SCU11 Δ*abrB*/Δ*upp*). All data were obtained from three independent experiments

*B. pumilus* BA06 contains a homolog gene of *aprE* of *B. subtilis* 168 [39], whose product has unique catalytic properties and potential use in the leather processing industry [39, 40]. The *aprE* of *B. subtilis* has been illustrated to be extensively regulated by several regulators (Fig. 2A) [41]. To determine if the regulatory functions of these regulators on *aprE* in *B. pumilus* were similar with those in *B. subtilis*, we disrupted the genes encoding SinR, Hpr, AbrB, DegS, and DegU in the strains of *B. pumilus* SCU11 or BA06.

For these strains, growth and extracellular alkaline protease activity were initially determined in LB broth (Fig. 2, Additional file 1: Fig. S1). At the early stage (within 12 h) of growth, there were no significant differences in cell growth among different strains, except for the *degS/U* mutant. However, discrepancies in the later stages became evident (Fig. 2B). At the time point of 36 h, the optical densities of the culture for strains BA06, SCU11, ∆*sinR*, ∆*hpr*, ∆*abrB*/∆*upp*, ∆*degS*, and ∆*degSU* were 6.2, 5.0, 4.2, 3.5, 2.2, 4.8, and 4.6, respectively. Meanwhile, extracellular protease activity was 71.6 U/mL, 342.4 U/mL, 400.0 U/mL, 615.8 U/mL, 817.6 U/mL, 0.05 U/mL, and 4.7 U/mL, separately. Disruption of *degSU* or *degS* led to a serious reduction of extracellular protease synthesis (Fig. 2C, Additional file 1: Fig. S1), implying that protease expression required DegU and its phosphorylation. SinR, Hpr, and AbrB exhibited a negative regulation on the protease expression, since their deletion mutants produced higher protease activity than the parent strains (Fig. 2C). However, deletion of *abrB* gene had the most significant contribution to enhance the extracellular protease synthesis, followed by *hpr* and *sinR*. In conclusion, these transcription regulators influence both growth and extracellular protease synthesis.

**Screening for hyper-active DegU mutants on*aprE*expression**.

**Fig. 3:**
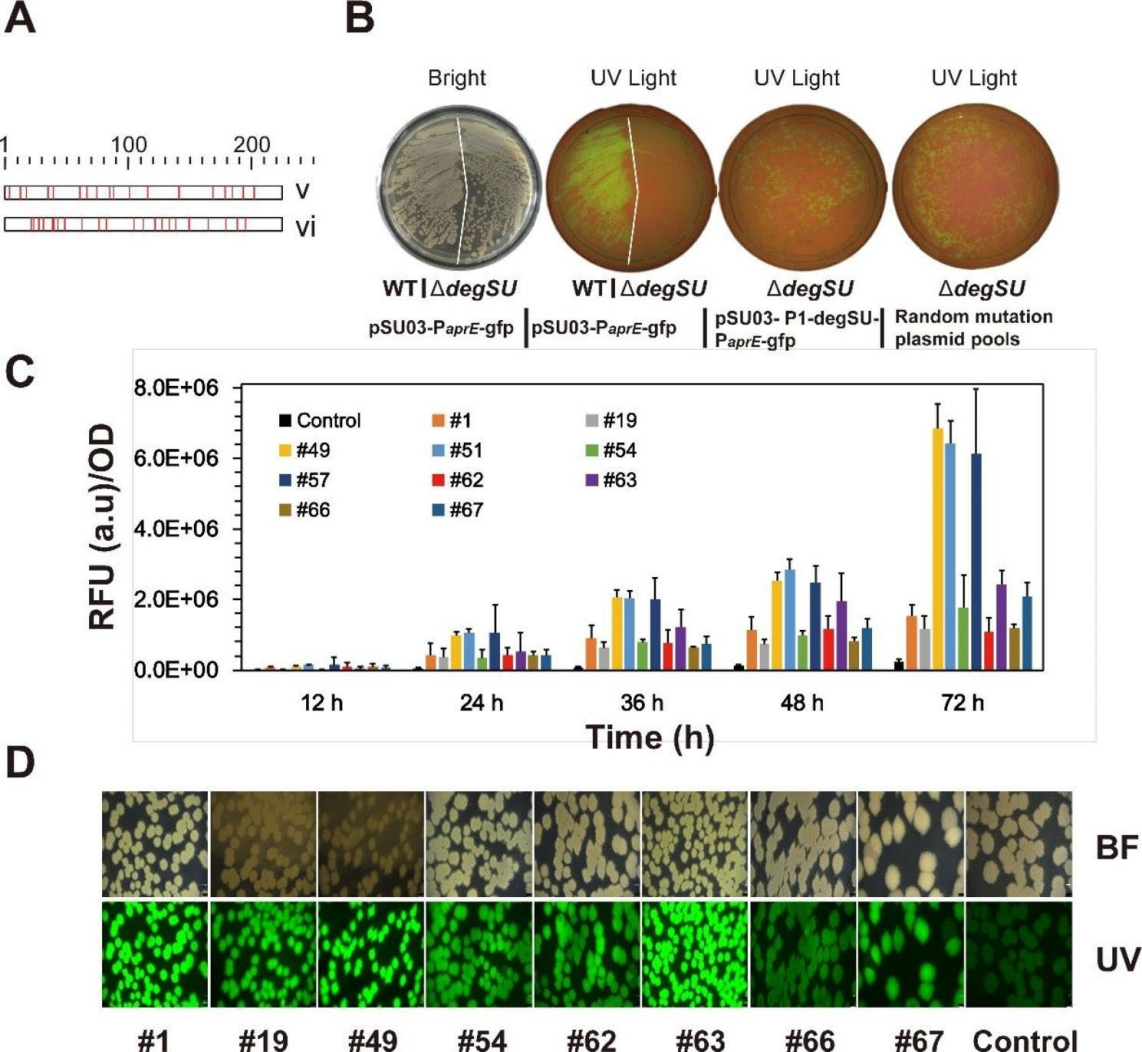
Screening of positive degU mutants in *B. subtilis*. **(A)** The mutation distribution across the DegU protein in two *degU* mutation libraries (lib v and lib vi). **(B)** Evaluation of *B. pumilus degSU* to drive *gfp* expression in *B. subtilis*. **(C)** The *gfp* expression of 10 selected transformants with highest RFU values over the time. **(D)** Illustration of nine recombined plasmids (excluding duplicates) extracted from the strains (subgraph C) and backcrossed to the “pure” chassis (*B. subtilis* Δ*degSU*). BF denotes the bright field scope, and UV denotes the ultra-violet scope. Control refers to the plasmid with the wild-type *degU.* All photos were taken under the exact same imaging conditions

We attempted to engineer hyperactive variants of DegU to boost AprE expression, as it has a significant impact on extracellular protease activity in *B. pumilus*. For this purpose, two *degU* mutation libraries were constructed using error-prone PCR in *E. coli*. DNA sequencing revealed that the nucleotide mutation frequencies in the mutagenesis pools were 6.08 bases/kb and 6.89 bases/kb, respectively. The mutation rates of transitions and transversions were similar. Figure 3 A shows the distribution of modified amino acid residues within DegU. Totally, more than 115,000 individual transformants were obtained.

We selected *B. subtilis* FDAARGOS 606 (referred to as *B. subtilis* 606 later) as the host strain for hyperactive DegU screening due to the poor transformability of the *B. pumilus* ∆*degSU* mutant. To eliminate the influence of endogenous DegSU in the host strain, we used the CRISPR/Cas9 method to inactivate the *degSU* operon via in-frame deletion. The resulting strain showed reduced extracellular protease activity (Additional file 1: Fig. S2), indicating successful inactivation of *degSU*. Therefore, we designed a fluorescent reporter system to screen the *degU* mutation library in *B. subtilis* 606. We firstly tested whether the *aprE* promoter was functional and driven by the DegSU of *B. pumilus* BA06 in *B. subtilis* 606. We introduced the plasmid pSU03-P_*aprE*_-gfp, which contains the reporter gene driven by the native *aprE* promoter of *B. pumilus*, into *B. subtilis* 606 wild type and its ∆*degSU* mutant. As expected, the reporter gene *gfp* was expressed only in the wild-type strain of *B. subtilis* 606, but not in the ∆*degSU* mutant (Fig. 3B). Furthermore, the expression of the reporter was restored in *B. subtilis* 606 Δ*degSU* when the *B. pumilus degSU* were introduced into the plasmid pSU03-P_*aprE*_-gfp (Fig. 3B). We concluded that foreign DegSU could drive *gfp* expression controlled by the *aprE* promoter in *B. subtilis* 606 ∆*degSU*.

The *degU* mutation libraries were transformed into *B. subtilis* 606 ∆*degSU* and more than 57,500 transformants were primarily examined by observing their fluorescent intensity on agar plates under UV irradiation. Subsequently, 67 clones with significantly improved fluorescence intensity were picked up and used to be further screened by quantitative fluorescence analysis through broth fermentation in 96-well plates. The selected clones exhibited a significantly increased fluorescent intensity of 2.5 to 35.2-fold compared to the control (the wild-type DegU). The fluorescence intensity of the best performing 10 clones was plotted against incubation time in Fig. 3C.

The recombinant plasmids were extracted from the 11 corresponding clones, including the control, and subjected to DNA sequencing. As summarized in Table 1, the mutation sites of 10 clones were identified. However, clones #49, #51, and #57 had identical mutation sites and were recognized as the same one. The H12L mutation type, previously reported to activate the exo-degreases in *B. subtilis*, was also detected in our DegU mutants 19B and 54 A [28]. Subsequently, eight DegU mutants with unique mutation sites were transformed back into *B. subtilis* 606 ∆*degSU* for further verification. These transformants exhibited a significantly higher fluorescence intensity than the control (Fig. 3D).

**Table 1:** Summary of selected DegU variants (Hy) with mutation sites

Name	Mutation sites	RFU (36 h)
1 A	E46D/D111V/P162L/V219E	15.23
19B	H12L/D87V/N90S/K118N/T144S/L177I/V201A	9.84
49 A/51A/57A	I8Y/T28S/G38R/Q77K/I165F	33.55^b^
54 A	H12L /V19A/E115K/V210A	11.86
62 A	L113F	11.28
63 A	Q13P/A121G/Q175H	18.91
66 A	N58Y/Q70L	9.56
67 A	A41T/S145C	12.62
Control	/	1.00^a^

^a^ the mean value of RFU readings of strains 49 A, 51 A, and 57 A, ^b^ Control was the recombinant plasmid hosting the wild-type degU, as shown in Fig. S3 VI.

**Construction of protease overproducer in*B. pumilus***.

A “push and pull” strategy was designed based on the regulation network of *aprE* to develop a protease overproducer in *B. pumilus*. This involved weakening negative regulation and elevating positive regulation simultaneously. The DegU(L113F) mutant was chosen to replace the native DegU in *B. pumilus* based on its simple mutation type and convenience of interpreting experimental results. The *B. pumilus* SCU11 ∆*hpr* mutant was chosen as the starting strain for further engineering due to the Δ*upp*/Δ*abrB* mutant resisted transformation operations which has the similar situation for *degS/U* deficient strains.

To integrate the mutation sequence encoding DegU(L113F) into the native *degU* locus in *B. pumilus* SCU11 ∆*hpr*, we used CRSPR/Cas9 genome editing. The resulting strain was assigned as 62 A and confirmed by PCR and DNA sequencing. Several phenotypes of 62 A were initially characterized. It was noticed that milk-hydrolytic halo on a 1% milk plate (Additional file 1: Fig. S4A), colony morphology (Additional file 1: S4B), cell mobility (Additional file 1: Fig. S4C), and flagella staining (Additional file 1: Fig. S4D, E) were altered in comparison with its parental strain, SCU11 ∆*hpr*.

**Fig. 4:**
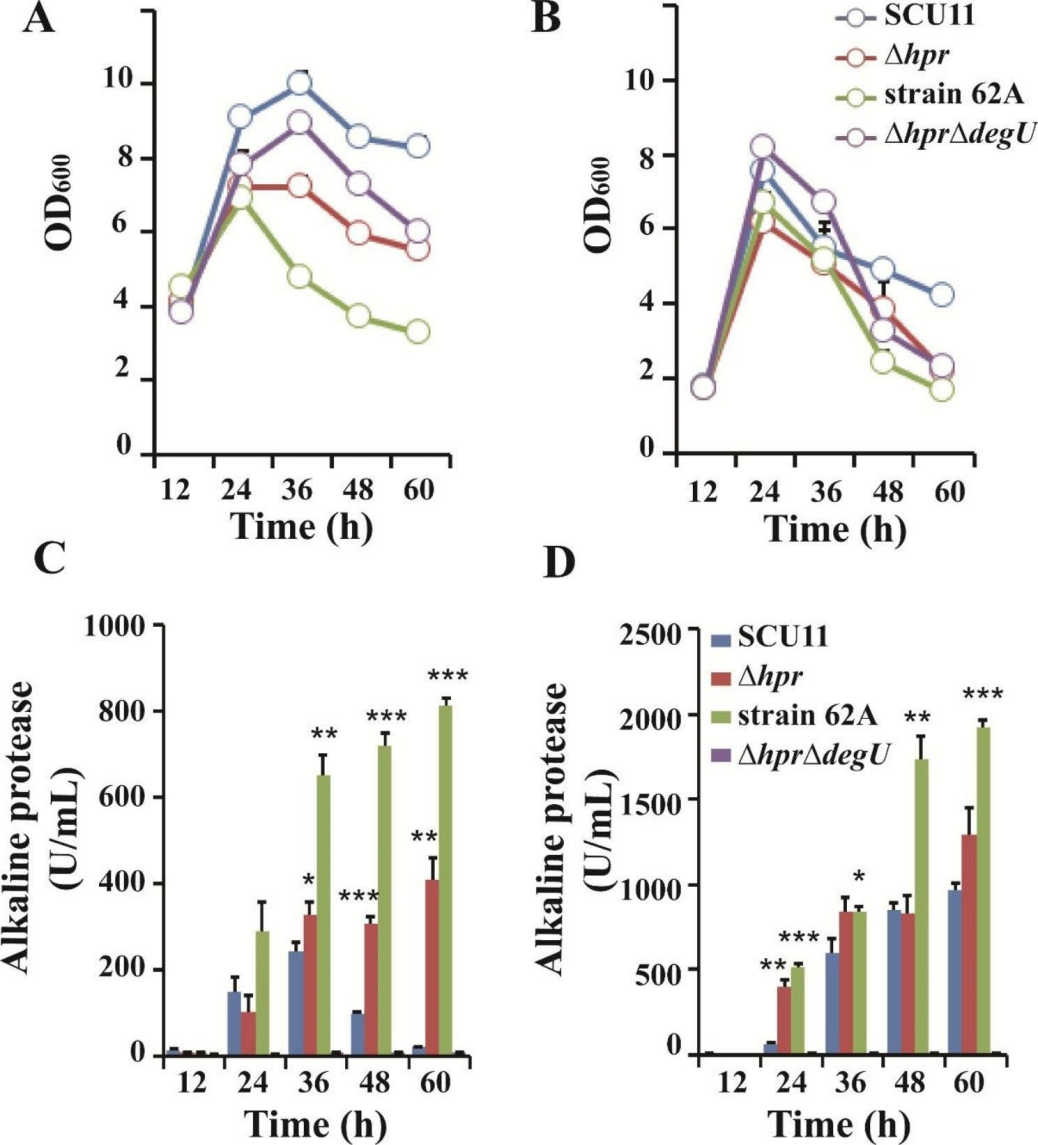
Growth and alkaline protease production of four *B. pumilus* strains. **(A)** The growth curves of four bacterial strains in LB (+ 0.2% gelatin) medium (37 °C, 200 rpm, aerobically). **(B)** The growth curves of four bacterial strains in fermentation medium (37 °C, 200 rpm, aerobically). **(C)** The extracellular alkaline protease activity of four strains in LB (+ 0.2% gelatin) medium (37 °C, 200 rpm, aerobically). **(D)** The extracellular alkaline protease activity of four bacterial strains in the fermentation medium (37 °C, 200 rpm, aerobically). The data were applied to student t-test with *p* < 0.05 (*), 0.01 (**) and 0.001 (***) compared with SCU11 at the same time points

We then performed fermentation experiments using laboratory and industrial medium to examine the protease biosynthesis of the four strains: SCU11, SCU11∆*hpr*, SCU11∆*hpr*/Δ*degU*, and 62 A in the shake flasks. In LB-based broth, the cell density of 62 A declined more than the other strains (Fig. 4A), and the extracellular protease activity increased rapidly within 36 h for all bacterial strains. After 36 h, the proteolytic activity of SCU11 decreased, which was significantly different from SCU11 ∆*hpr* and 62 A. The maximum extracellular protease titer of 62 A approached 812.5 ± 15.9 U/mL at 60 h, which was 3.3-times and 1.0-times higher than peak values of SCU11 and SCU11 ∆*hpr*, respectively (Fig. 4C). The cell growth of these strains was almost consistent in the fermentation medium (Fig. 4B), but the proteolytic activity was further improved compared to that in LB medium for all tested strains. The strain 62 A achieved a maximum protease activity of 1926 U/mL at 60 h (Fig. 4D).

### Transcriptome analysis of high-yield strain 62 A

**Fig. 5:**
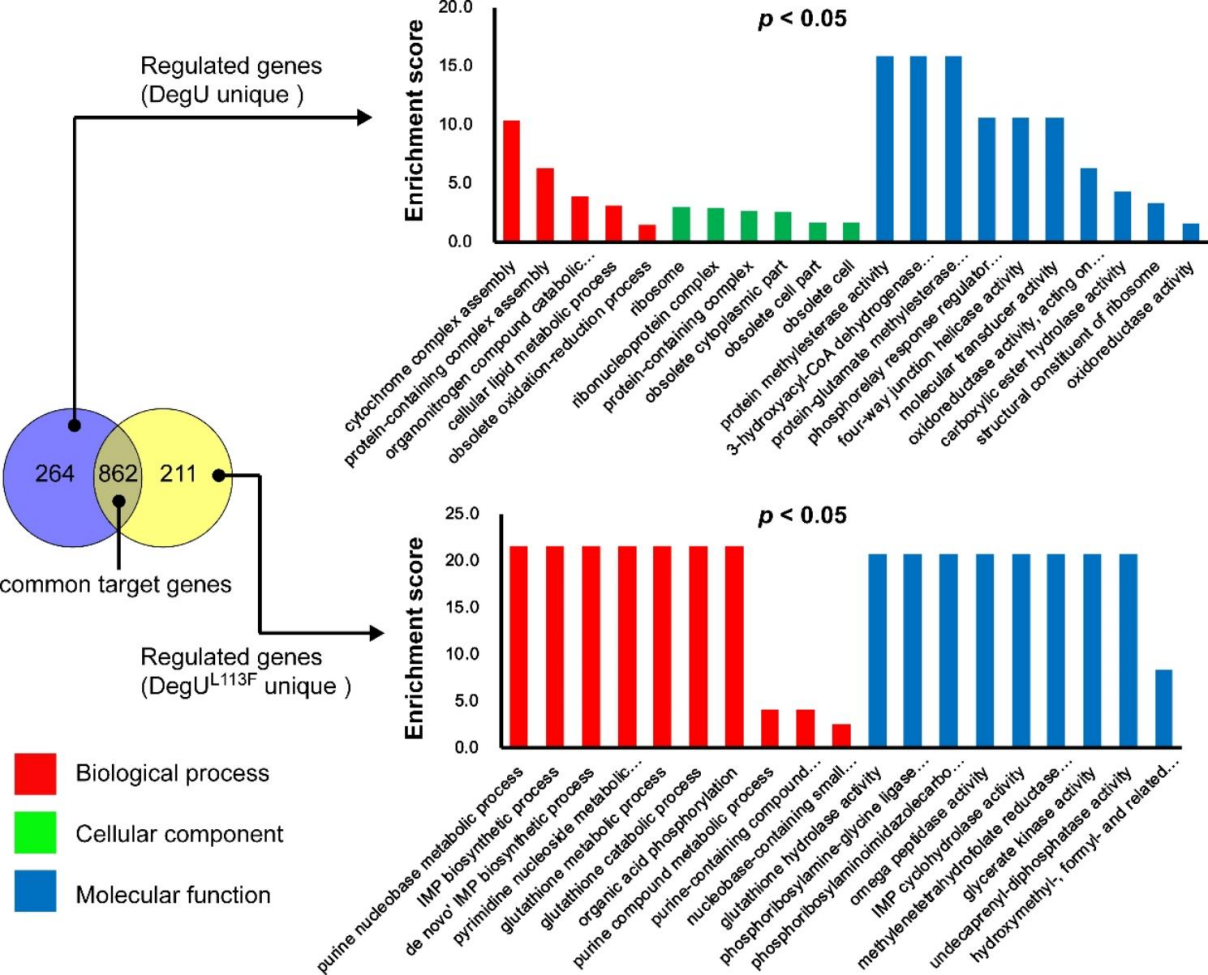
Veen diagram and GO enrichment of DEGs in ∆hpr mutants and high-yielding strains at the time point of 24 h. The two gene sets above were the differentially expressed genes obtained from the mutant ∆*hpr* and the strain 62 A related to the double knockout strain, respectively

To evaluate the metabolic perturbations introduced by the engineered transcription factor DegU(L113F), we conducted a comparative transcriptome analysis of three strains of SCU11 Δ*hpr*, SCU11 Δ*hpr*/Δ*degU*, and 62 A, which were cultivated in LB medium supplemented with 0.2% gelatin. We identified 1073 and 1126 differentially expressed genes (DEGs) in 62 A and SCU11 Δ*hpr*, respectively, at the 24-hour time point relative to the control strain (SCU11 Δ*hpr*/Δ*degU*) (Additional file 2: Table S1, Table S2). Among the regulated genes, 862 DEGs were commonly regulated by both the native DegU and its mutant DegU(L113F), sharing almost 65% of the all regulated genes. The gene ontology enrichment analysis of these specific DEGs demonstrated that DegU(L113F) gained several additional targets (211 genes), which were highly conserved in the metabolism of purine compounds, while losing some cellular functions related to cell morphogenesis (Fig. 5) (Additional file 2: Table S3).

Given that *B. pumilus* produces several commercially important extracellular proteases, we examined the expression levels of the eight main extracellular protease genes (Table 2). Interestingly, all eight protease genes were almost down-regulated in SCU11 Δ*hpr*/Δ*degU*, highlighting DegU’s global regulation role in extracellular degradative enzymes [42, 43]. AprE was the most abundant extracellular protease in terms of transcription levels, which was in agreement with previous studies that AprE accounts for more than 70% of extracellular protease activity in *B. pumilus* [37]. Notably, the transcription level of *aprE* in 62 A was significantly higher than that in the Δ*hpr* mutant (log_2_FC = 1.06, *p*-value = 0.007) at the 24-hour time point. Furthermore, *aprE* expression was highly maintained in strain 62 A at the 36-hour time point compared to SCU11 Δ*hpr*. However, the genes encoding other proteases did not show significant changes in transcription in strain 62 A compared to SCU11 Δ*hpr*.

**Table 2:** Relative expression level (RPKM) of eight protease and four regulator genes of the three strains across three time points

Gene Name	Gene_ID	Length (bp)	Protein	∆*hpr*			Strain 62 A			∆*hpr* ∆*degU*		
				12 h	24 h	36 h	12 h	24 h	36 h	12 h	24 h	36 h
*wpr*	RS01295	2667	Cell wall-associated protease	1417	423	373	2003	877^a^	469	1003	510	21
*epr*	RS01360	1632	Minor extracellular protease	56	28	15	57	42	16	52	48	11
*aprE*	RS04960	1146	Subtilisin E (AprE)	1442	47,486	13,850	4803^a^	100,190^a^	73,727^a^	150	1285	1896
*isp*	RS06215	960	Major intracellular protease	157	12,688	1943	889	15,456	2788	85	1339	1444
*bpr*	RS07255	4320	Bacillopeptidase F	113	1177	211	293	1436	243	12	100	51
*aprX*	RS08225	1326	Serine protease AprX	3	151	34,203	5	368^a^	11,140	3	18	6666
*aprN*	RS10535	1131	Subtilisin NAT	36	19	13	45	23	9	11	14	20
*vpr*	RS17695	2424	Minor extracellular protease	3180	1465	860	2806	882	418	472	777	284
*degS*	RS16465	1176	Sensor histidine kinase	78	78	35	92	95	143	58	54	21
*degU*	RS16460	690	Two-component system regulator	613	790	230	817	1112	1181	0	0	0
*sinR*	RS15940	471	Anti-repressor SinI	49	29	20	23	39	18	18	48	23
*abrB*	RS00080	291	AbrB family DNA-binding protein	1496	153	158	1525	263	547	3723	578	81

^a^ indicating the significant fold-change with |log_2_FC| > 1.00 and *p* < 0.05 (compared with ∆*hpr* mutant)

### Structural modelling and molecular dynamics simulation of DegUs

**Fig. 6:**
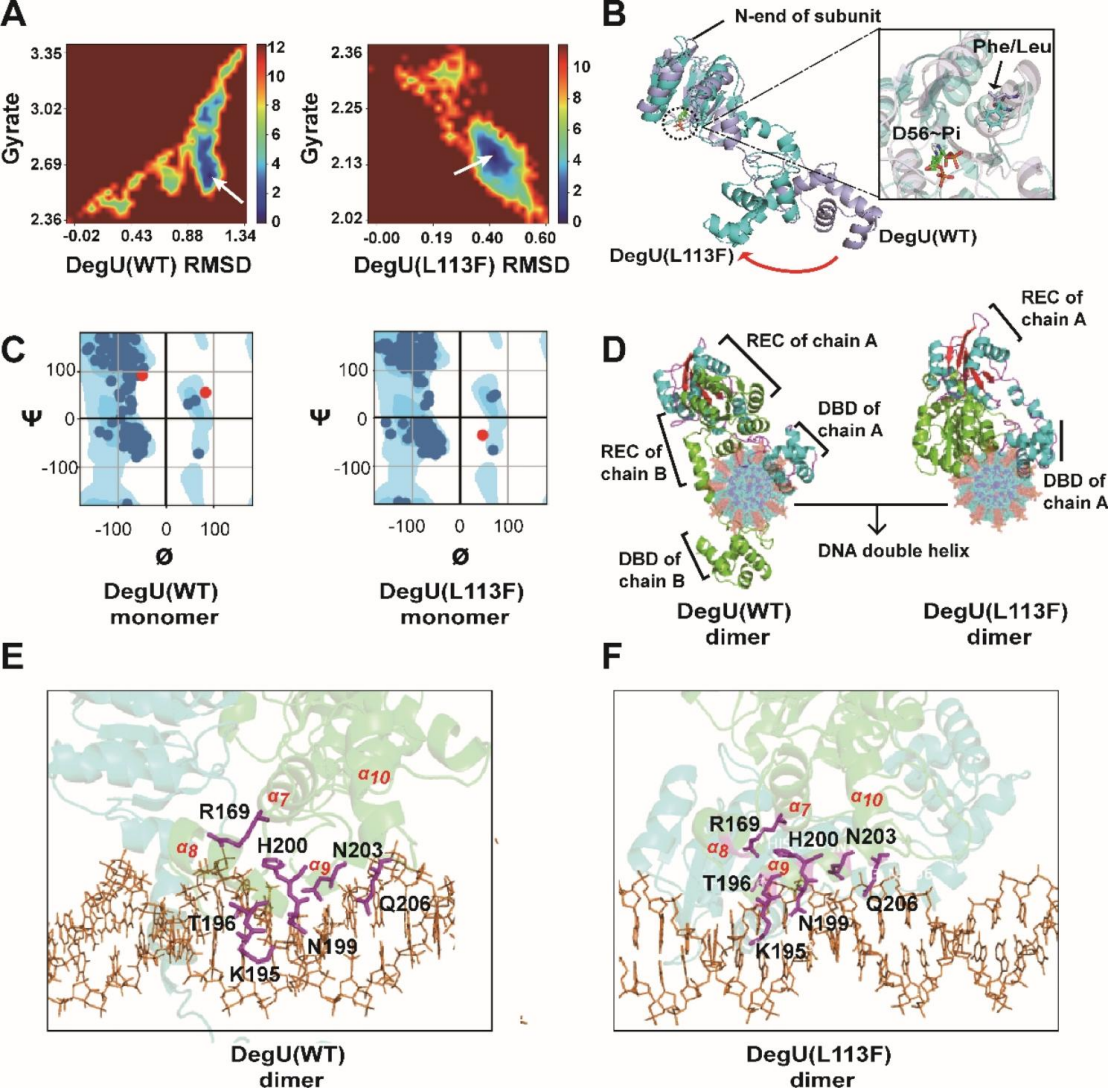
The molecular dynamic simulation of DegU(WT), DegU(L113F) and docking on *aprE* promoter. **(A)** The Gibbs energy landscape of DegU(WT) and DegU(L113F). A lower Gibbs energy reflects a more stable configuration state, and the global optimal configuration of each protein was highlighted by a white arrow. RMSD means root mean square deviation. **(B)** Overlay of protein structures of DegU(WT) and DegU(L113F) and the details around the phosphate pocket. **(C)** Ramachandran plot of DegU(WT) and DegU(L113F), where ø and ψ were the two dihedral angles formed by the C_*α*_ atoms of the main chain. Each dot represented one residue of proteins. The red dots represented residues possessed an undesirable dihedral conformation. The plot was generated by PyRAMA software. **(D)** DNA binding by DegU(WT) and DegU(L113F). The pictures were obtained from the top-down view of the DNA spindle. **(E)** Interactions of the R169, K195, T196, N199, H200, N203 and Q206 residues (purple sticks) with dsDNA (orange lines and base rings) in the model of a complex between the DegU(WT) dimer (green and cyan transparent ribbons) and dsDNA. **(F)** Interactions of the R169, K195, T196, N199, H200, N203 and Q206 residues (purple sticks) with dsDNA (orange lines and base rings) in the model of a complex between the DegU(L113F) dimer (green and cyan transparent ribbons) and dsDNA. The structures were aligned and plotted using PyMol software

The amino acid sequence of the native DegU in *B. pumilus* BA06 underwent analysis by submitting it to NCBI, revealing that the organization of DegU comprised an *N*-terminal REC domain and a *C*-terminal DNA-binding domain (DBD) with a typical helix-turn-helix (HTH) structure [44]. We performed molecular dynamic simulations to evaluate the molecular behaviors of DegU(WT) and DegU(L113F). The Gibbs energy landscape of proteins (Fig. 6A) showed that DegU(L13F) obtained a concentrated optimal conformation compared to native DegU during the simulation, suggesting that the protein DegU(L113F) had a faster route of global energy minimization (fast folding). On the other hand, the DegU(L113F) mutant had a smaller gyrate radius than its cognate protein (0.34 nm for mutant versus 0.99 nm for DegU), indicating a more compact protein structure (less exposure). Figure 6 C showed the Ramachandran plot of DegU(WT) and DegU(L113F), respectively, which demonstrated the rationality of the models. Overlaying the corresponding optimal configurations of DegU(WT) with DegU(L113F), it was observed that the region adjacent to position L113 was not significantly altered while the tail of DegU(L113F) rotated almost 90 ° toward the dimer interface (Fig. 6B). These results indicated that the L113F mutation could influence the overall structure of DegU through long-range effects [45]. Furthermore, the DegU(L113F) dimer possessed more hydrogen bonds and hydrophobic effects than DegU(WT) (Additional file 1: Fig. S5).

Our preliminary research revealed that the recombinant DegU protein can be bound to a specific core sequence, roughly 50 nucleotides, within the *aprE* promoter (data not shown), which was used to be docked to the dimer of DegU and its mutant (L113F). It was shown that the DegU dimer formed a “clamp-like” structure that ‘rides’ on the DNA double helix, and the HTH domain attached to the DNA major groove simultaneously (Fig. 6D). Furthermore, the DegU(L113F) dimer collapsed toward the interface of protein-DNA complex. Although there was a dramatic general structural alteration existed between DegU and its mutant, the critical four α-helixes (α_7_ ~ α_10_) in the HTH domain of chain A during DNA recognition did not varied (Fig. 6E, F) [44]. Residues R169, K195, T196, N199, H200, N203, and Q206 of chain A were shared residues within α_7_, α_9_ of DegU and DegU(L113F). The importance of residues R169, T196, and H200 was previously validated [19, 29]. The positively charged K195 residue from the α_9_ helix was located in the positive patch and interact with the DNA bases in the major groove of dsDNA in the complex. The neutral hydrophilic residues N199, N203, and Q206 at the periphery of the positive patch of the α_9_ helix also seems to function as a DNA base binder. More residue information can be obtained in Additional file 1: Fig. S6.

## Discussion

Shimane and Ogura (2004) used alanine scanning mutagenesis to analyze the DegU helix-turn-helix region in the DNA-binding domain to identify 27 variants, of which five mutants (N183, I192, T196, H200, and L205) exhibited reduced DNA binding activity and severe reductions in the expression of the *aprE* and *comK* genes [29]. In addition, several DegU variants of H12L, T98I, E107K, and V131L were identified as hyper-secretion DegU mutants [17, 19, 20, 24, 28]. The aforementioned molecular docking results revealed that T98, T196, H200, and L205 were critical residues at the factor-ligand interface, while N183 and I192 were proximal to these key residues. H12 was critical for dimerization, whereas E107 and V131 were not located on protein surfaces or at the factor-ligand interface (Additional file 1: Fig. S6). It should be noted that L113 was involved in mediating the hydrophobic effect between the subunits of the DegU dimer. The L113F alteration produced spatial resistance introduced by the benzene ring can trigger the rearrangement of DegU protein and hence may change its regulation behaviors. Furthermore, the intrinsic properties (fast folding and less exposure) of DegU(L113F) may decrease the chance of proteolysis by the ClpCP degradation system (DegU was rapidly degraded in *B. subtilis* wild type via ClpCP-Spx system) and lead to higher DegU-Pi levels [13, 46].

In general, from the distribution of mutation sites across the DegU sequence, the reported down-regulation mutations were commonly found in the DNA-binding domain (except for D54, which was the phosphorylation site), while up-regulation mutations are dispersed and varied (Additional file 1: Fig. S6). In this study, the fact that these identified hyper-active DegU candidates that affect DegU’s activity were disorganized throughout the DegU sequence seemed to obey the above rule (Additional file 1: Fig. S6).

In conclusion, despite the high conservation of DegU sequences in the *Bacillus* genus (Additional file 1: Fig. S7), DegU’s structure also exhibited a high degree of plasticity, thereby providing a foundation for utilizing DegU as a potential target for genetic modifications for multiple environments (gTME) and vice versa.

DegU was a global regulatory system that affects the expression of over 100 genes in *B. subtilis*, all of which were involved in various metabolic pathways with diverse functions [47–51]. The multiple functions of DegU as a regulator were also demonstrated in the recent study on the lichenysin biosynthesis regulation network in *B. licheniformis* [52]. In the case of *B. pumilus*, our data show that deletion or mutation of DegU led to changes in transcriptional levels of more than 1,000 genes (Additional file 2 Table S1 and Table S2), suggesting that it may also play such multiple regulatory roles in this species.

Analysis of gene expression data shows that the transcription of the DegU gene was roughly correlated with that of the AprE gene across three sampling points. However, at the time points of 12 and 24 h, the fluctuation in DegU gene transcription levels between strain 62 A and the ∆*hpr* mutant was less than 40%, while AprE transcription levels in strain 62 A were 3.28-fold and 2.08-fold higher than those in the ∆*hpr* mutant, respectively. At 36 h, the fact that transcription levels of *aprE* decrease dramatically in the ∆*hpr* mutant but not in strain 62 A (Table 2) suggested that the DegU mutant inspired positive autoregulation loop of DegU synthesis (Fig. 2A), and therefore improved *aprE* transcription even in the later cell phase, as previously observed in *B. subtilis* [25]. In support of this, the phosphorylated form of DegU has been shown to act as a direct activator of the *pgs* operon in *B. subtilis* [53, 54]. Similarly, the transcriptome data in this investigation showed that poly-gamma-glutamate synthases (*pgsB* and *pgsC*) were significantly up-regulated (log_2_Fold Change = 2.86, 2.47; 2.139, 1.827, 12 and 24 h, respectively) compared to the ∆*hpr* mutant (see Additional file 2: Table S4, 5), indicating that DegU(L113F) had a stronger ability to activate the transcription of certain target genes.

In addition to regulating expected extracellular protease, our data also showed that the DegU mutant exhibits multi-pathway regulation properties (see Additional file 2: Table S4 ~ 9) and this effect manifested itself in disables in regulating multicellular behaviors [55–57]. One of the characteristics that represent these multicellular behaviors was bacterial motility. The strain 62 A supported swimming motility but abolished swarming motility (Additional file 1: Fig. S4C), while DegU32(Hy) mutation in *B. subtilis* causes a complete loss of both motility [21, 58]. DegU(L113F) in SCU11 displayed a unique function that differs from both DegU(WT) in SCU11 and DegU32(hy) in *B. subtilis*. Previous research has shown that swarming motility was dependent on the expression of the exoprotease, Epr (low DegU-Pi) [59]. Furthermore, using transposon mutagenesis, combined with high-throughput sequencing, Sandra Sanchez et al. (2022) identified genes that were essential for swarming motility in *B. subtilis*, clustered into two classes, called “*swr*” and “*fla*” and showed that the flagellar biosynthesis was defective and swarming motility was lost, while swimming motility remained unaffected [60, 61]. The transcriptome data presented in Table 2 have indicated that the DegU mutation (L113F) did not affect the expression of *epr* in *B. pumilus*. KEGG analysis of genes in strain 62 A (related to ∆*hpr* mutant, 12 h, Additional file 2: Table S10) indicated that flagellar assembly proteins and genes related to bacterial chemotaxis-related genes were highly enriched. These genes converged to the ‘*fla*’ class and correlated with swarming activity (Additional file 1: S8Fig. , 9). Due to the difficulty in counting the exact number of flagella per cell in flagella-staining, bacteria with distinct flagella (more than 3 flagella per cell) were counted. However, the flagella-staining results showed that the ratios of cell with flagella between ∆*hpr* mutant and strain 62 A remained unchanged (**Fig. S4**E). But the average number of flagella per cells in strain 62 A were below than that of the ∆*hpr* mutant based on visual inspection (Additional file 1: Fig. S4D). Besides, former study had shown that the disruption of the *hpr* gene reduces cell motility as it reduced flagella formation [62] and so caused the flagellar number in strain 62 A (∆*hpr* genetic background) below a certain threshold needed for swarming motility which was observed in *B. subtilis* [63]. Recently, it has been demonstrated that very low concentrations of DegU-Pi activate swarming motility, whereas high concentrations of DegU-Pi completely abolish it [64]. It was speculated that the genes related to up-regulated motility (12 h) mentioned above were owing to the initial high levels of unphosphorylated DegU(L113F) synthesis (active *fla-che* operon transcription needed the unphosphorylated DegU) during the early log phase (before 12 h) and inhibited rapidly by higher levels of phosphorylated DegU(L113F) levels later (at 24 h, as synthesis of AprE began and active *aprE* transcription depended on high concentrations of phosphorylated DegU) (Fig. 4C).

To sum up, compared with native DegU, DegU(L113F) mutant accumulated rapidly by resisting degradation and increasing positive regulation of synthesis and, as a consequence, enhanced regulation of certain genes, such as *aprE*, *pgsB* and *pgsC* et al., on the one hand; reduced regulation of some genes such as *fla-che* operon and so on.

Additionally, the classic gTME approach has two limitations: firstly, the screening and selection was performed in the wild-type background and mutant proteins with good performances may be discarded while in competition with the wild-type version of protein; secondly, the transformation efficiency limits the application of the method when applied to undomesticated nature microorganisms with lower genetic manipulation capability. Our work offers a new path for solving both of these problems.

## Conclusions

In this study, we explored the utilization of gTME concerning the global transcription factor, DegU, in *B. pumilus* for alkaline proteases production.We increased alkaline protease activity by 80% by disrupting *hpr* in *B. pumilus* SCU11, and further increased by an additional 50% by integrated with DegU(L113F). Our findings have important implications for the industrial production of AprE by *B. pumilus*, and DegU(L113F) may be applicable in other *Bacillus* species.

## Materials and methods

### Strain, plasmid, culture, and materials

In this study, a variety of bacterial strains and plasmids were utilized (Table 3). *Escherichia coli* DH5α was selected for vector construction and was grown in Luria-Bertani (LB) medium, containing tryptone (10 g/L), yeast extract (5 g/L), and NaCl (5 g/L). *B. pumilus* BA06 and its derivatives were cultivated aerobically at 37 °C in LB medium. Antibiotics, including kanamycin (25 µg/mL), chloramphenicol (10 µg/mL), erythromycin (5 µg/mL), and ampicillin (50 µg/mL), were added to the media as needed. Gene deletion in *B. pumilus* BA06 was carried out by using temperature-sensitive plasmid pUCE^ts^, while gene disruption in *B. pumilus* SCU11 and *B. subtilis* was performed by using plasmid pJOE8999.1. Gene overexpression and functional screening was carried out by using the modified expression plasmid pSU03-AP.

The fermentation medium for alkaline protease contains soybean peptone (15.0 g/L), yeast extract (3.0 g/L), NaH_2_PO_4_ (0.4 g/L), K_2_HPO_4_ (4.0 g/L), potato extract (extracted from 200 g of fresh potato per one litre of culture medium) and CaCO_3_ (3.0 g/L). The final pH was adjusted to 7.4 ± 0.2 (25 °C).

The main primers used in this study were present in Additional file 2: Table S11.

**Table 3:** The bacterial strains and plasmids used in this study

Strains and Plasmids	Descriptions	Source
**Strains**		
*B. pumilus* BA06	The raw strain isolated for dehairing alkaline protease production	[36]
*B. pumilus* SCU11	AprE high-yielding strain, mutagenesis from BA06	[40]
*B. pumilus* BA06 Δ*degS*::Cm^R^	Disruption of *degS* gene	This study
*B. pumilus* BA06 Δ*degSU*::Kan^R^	Disruption of *degSU* gene	This study
*B. pumilus* SCU11 Δ*sinR*	Disruption of *sinR* gene	This study
*B. pumilus* SCU11 Δ*hpr*	Disruption of *hpr* gene	This study
*B. pumilus* SCU11 Δ*upp*/Δ*abrB*	Disruption of *abrB*/*upp* gene	This Lab^a^
*B. pumilus* SCU11 Δ*hpr* Δ*degU*::*degU(L113F)*	in situ replacement of the *degU* gene with a mutant *degU* in a Δ*hpr* mutant	This study
*B. subtilis* FDAARGOS_606 (Δ*degSU*::*comK*)	Disruption of *degSU* gene and integrate an inducible *comK* element in chromosome	This study
*Escherichia coli* DH5α	DNA cloning host	This Lab
**plasmid**		
pUCE^ts^	Temperature-sensitive plasmid used for gene disruption in *Bacillus*	[62]
pJOE8999.1	CRISPR/Cas9 genome editing in *Bacillus*	[65]
pUCEts-degS::Cm^R^	Temperature-sensitive plasmid used for *degS* disruption	This study
pUCE^ts^-degSU::Kan^R^	Temperature-sensitive plasmid used for *degSU* disruption	This study
pJOE8999.1-∆*sinR*	For *sinR* disruption	This study
pJOE8999.1-∆*hpr*	For *hpr* disruption	This study
pJOE8999.1-degU(L113F)-M	Replacement of *degU* by *degU*(L113F) in *B. pumilus*	This study
pSU03-AP	*Bacillus*-*Escherichia* shuttle vector harboring *aprE* gene.	[66]
pSU03-P_*aprE*_-gfp	*Bacillus-Escherichia* shuttle vector Reporter gene driven by P_*aprE*_ of *B. pumilus*	This study
pSU03-P_1_-degSU-P_*aprE*_-gfp	Reporter gene driven by P_*aprE*_ of *B. pumilus*, foreigner *degSU* gene derived from *B. pumilus* and driven by *B. subtilis* P1 promoter. Used for library preparation and fluorescence quantification baseline	This study

^a^ The *upp* gene, which encodes uracil phosphoribosyl transferase, was utilized as a counter-selectable marker for achieving markerless gene deletion. According to our unpublished data, the usage of *upp* gene did not affect the production of AprE, and had a negligible impact on growth and colony morphology.

### Plasmid construction and DNA manipulation

The study followed the established protocols of DNA manipulation [67]. As an example, pJOE8999.1-degU(L113F)-M was constructed as follows. Details of additional plasmids and related information were available in Additional file 1: **Fig. S3**.

Two DNA fragments (LH and RH) flanked the *degU* gene were amplified by High-fidelity 2 × phanta max Master mix (Vazyme, China) with the primer pairs (degU-LF_xma1/degU-LR and degU-RF/degU-RR_xma1) and using genomic DNA of *B. pumilus* BA06 as template. The mutation sequence of *degU* (L113F) was amplified from the screened plasmid pSU03-P_1_-degSU-P_*aprE*_-gfp (harboring the L113F mutation) with primer pair DegU(hy)_F/R. Overlap-PCR was used to assemble the LH and *degU*(L113F) fragments, which was then cloned into pMD19-T vector. The obtained plasmid was denoted as pMD19-LH-degU(L113F). We altered the sequence within the *degU* coding area targeted by the small-guide RNA to avoid an autoimmune effect, using 366 F/R primers and pMD19-LH-degU(L113F) as a template. The resulting plasmid was designated as pMD19-LH-degU(L113F)-M. Primers benchling 366 F/R was used to insert sgRNA into plasmid pJOE8999.1 via inverse-PCR, creating pJOE8999.1-M366. The fusion fragment (LH-degU(L113F)) was amplified with primers degU-LF_xma1/DegU-RR_xma1 with pMD19-LH-degU(L113F)-M as template. The plasmid pJOE8999.1-M366 was linearized with *Xma* I. Hence, the fusion fragment (LH-degU(L113F)-M, RH fragment, and the linearized pJOE8999.1-M366 were mixed for multi-fragment recombination by using the ClonExpress Ultra One Step Cloning Kit (Vazyme, China), resulting to obtain the anticipated plasmid pJOE8999.1-degU(L113F)-M.

### Plasmid transformation

To transform the plasmids into *E. coli*, chemical competent cells with an ordinary heat-shock method was performed followed well-known protocols [67]. The high-osmolarity electroporation protocol was adopted to transform the plasmids into *B. pumilus* [68]. Transforming the plasmids into *B. subtilis* was followed by an electroporation procedure developed by Zhang et al. (2011) with minor modification.

### Screening of the gene deletion strains

For the temperature-sensitive plasmids, positive transformants were spread onto LB (+ 1% milk) plates and cultured overnight at 37 °C. A single colony with a clear hydrolysis halo was selected and inoculated into 4 mL of LB medium (without antibiotics). The culture was initially incubated at 30 °C for about 2 h, and then transferred to continue incubating at 42 °C for 6 h. The resultant culture was diluted to an appropriate concentration and spread onto LB plate amended with 5 µg/mL chloramphenicol. The plates was incubated at 42 °C until formation of colonies, which were picked up and spotted onto the fresh plates with addition of 5 µg/mL erythromycin and 5 µg/mL chloramphenicol. The colonies that only grew on chloramphenicol plates were selected for colony PCR inspection to verify the gene disruption.

For CRISPR-based genome editing, positive transformant colonies were inoculated into 4 mL of LB medium amended with 20 µg/mL kanamycin and 0.2% mannose, which was incubated overnight at 30 °C with shaking at 200 rpm. The culture was diluted and spread on LB plates containing 0.2% mannose, which was incubated at 42 °C until individual colonies formed. The colonies were picked up to verify the gene deletion by the colony PCR.

### Random mutagenesis and screening of functional DegUs

Error-prone PCR was employed to introduce mutations in the DegU gene of *B. pumilus*. The PCR reaction was carried out in a total volume of 50 µL containing 2.5 U rTaq polymerase, 2.0 µL dNTP mix (2.5 mM each), 0.4 µM each of primers libMut_F and libMut_R, 10 ng of template DNA (pSU03-P_1_-degSU-P_*aprE*_-gfp), and various concentrations of Mn^2+^ and dATP (0.2/0.25 mM and 0.4/0.5 mM, respectively). The amplification condition was as follows: initial denaturation at 98 °C for 120 s, followed by 45 cycles of 10 s at 98 °C, 30 s at 60 °C, and 60 s at 72 °C. Two sets of mutagenesis pools were obtained from different combinations of Mn^2+^ and dATP concentrations. A small portion of each pool (50 ng roughly) was cloned into the pMD19-T vector to assess the mutagenesis efficiency.

After linearizing plasmid pSU03-P_1_-degSU-P_*aprE*_-gfp through PCR with primers pSU03-F/R and removing residual template DNA using *Dpn* I, the plasmid backbone was ligated with the *degU* mutagenesis pools by in vitro recombination using Vazyme’s One-step Cloning kit. The resulting product were transformed into *E. coli* DH5α to obtain two *degU*-mutation gene libraries (lib. v and lib. vi).

The plasmid DNAs derived from two libraries were electroporated into 80 µL of *B. subtilis* FDAARGOS 606 Δ*degSU* cells, which were spread on LB plate supplemented with 20 µg/mL kanamycin and incubated at 37 °C for about 48 h. The fluorescence intensity of each colony was then assessed under the UV light. Only the colonies exhibiting brighter fluorescence were selected and transferred onto the fresh plates for verification. Subsequently, individual colonies were picked up and inoculated into 96-well plates (1.8 mL LB per well). After incubating at 37 °C reaching the sampling time point, 100 µL culture was sampled to determine the cell density and fluorescence intensity.

### Construction of protease overproducer

The plasmid pJOE8999.1-degU(L113F)-M was introduced into the *B. pumilus* ∆*hpr* mutant by electroporation. The *B. pumilus* strain with replacement of the native *degU* with its mutation sequence encoding DegU(L113F) was screened following the procedure as described above.

### Fluorescence assays

Fluorescence intensity of sfGFP (excitation at 485 nm and emission at 510 nm) and cell density at OD_600_ were measured on the Biotek Synergy H1 microplate reader. Relative fluorescence intensity (RFU/OD) of the trials were calibrated according to following Eq. 1.

1 $$\left[\frac{\text{R}\text{F}\text{U}}{\text{O}\text{D}}\right]car = \frac{Ft-Fc}{ODt-ODc}$$

Where the Fc represents the background fluorescence of the bacterial culture of B. pumilus hosting a native degU pSU03-P1-degSU-PaprE-gfp; ODc represents the background OD_600_ of the medium. F_t_ and OD_t_ indicate fluorescence intensity and cell density of the tested bacterial strain hosting a given degU mutant.

### Fed-batch fermentation and extracellular alkaline protease assay

The seed culture was grown in 50-mL shake flasks containing 10 mL of LB broth at 30 °C by shaking at 220 rpm for 16 h. Subsequently, the seed culture was adjusted to an OD_600_ of approximately 1.00 and then inoculated at a 1% ratio into 50 mL of fermentation medium and LB in the baffled 250-mL shake flasks. The fermentation was proceeded at 37 °C with shaking at 220 rpm.

Protease activity was evaluated as described by Huang et al. (2003). A 1-mL aliquot of the enzyme solution diluted in 50 mM sodium borate-NaOH buffer (pH 9.6) was mixed with 1 mL of 2% casein substrate in a test tube and incubated at 50 °C for exact 10 min. Immediately, the caseinolytic reaction was stopped by adding 2 mL of 0.4 M trichloroacetic acid, and the mixture was then filtered through a filter paper. To 1-mL filtrate was added with 5 mL of 0.4 M Na_2_CO_3_ and 1 mL of Folin-hydroxybenzene solution. The mixture was incubated for 20 min at 40 °C. The absorbance was measured at 680 nm. The amount of enzyme required to liberate 1 µg tyrosine was defined as one unit of caseinolytic activity.

$${\text{Extracellular protease activity (U/ml) = }}\Delta {\text{O}}{{\text{D}}_{680{\text{nm}}}} \times {\text{N}} \times {\text{40}}\left( {{\text{N = the dilution factors}}} \right)$$

### Comparison transcriptome analysis of the bacterial strain harboring DegU(L113F) and related strains

Three *B. pumilus* strains of SCU11 Δ*hpr*/Δ*degU*::*degU*(L113F) (referred to as 62 A later), SCU11Δ*hpr*, and SCU11 Δ*hpr*/Δd*egU* were cultured in LB medium supplemented with 0.2% gelatin at 37 °C with shaking at 200 rpm. The cell samples were collected at 12, 24, and 36-hour by centrifugation at 4 °C and 13 000 × g for 4 min, which were immediately immersed in liquid nitrogen. RNA extraction and sequencing were outsourced to Beijing Novogene Biotechnology Co., Ltd. The final dataset size was about 2.0 GB per sample after data cleaning. The RNA-seq reads were then mapped to the reference genome (NZ_CP038517.1), and annotated using HTSeq and Bowtie2. Subsequently, the differentially expressed genes (DEGs) were screened out with a *p*-value < 0.05 and |1og_2_FoldChange| > 1.0 by using DESeq2 v1.20. Finally, GO enrichment analysis was conducted for the DEGs using TBtools software.

### Molecular dynamic simulation of DegUs monomer behavior

Using response regulator protein VraR (PDB ID: 5hev) as the template, homologous modeling of both wild-type DegU protein and its L113F mutant were performed on Swiss-Model (https://swissmodel.expasy.org/interactive). Subsequently, D56 in both proteins were phosphorylated in silico using Vienna-PTM [69, 70] and all ions were ionized prior to subjecting the proteins to 20 ns molecular dynamics (MD) simulation using GROMACS software [71]. The structure modeling of the resulting DegU and its mutant was dimerized, and hence docked to a specific core sequence within the *aprE* promoter (5’-ATTCCAAGCGACTTAATTCCCTATTTTTCGCTAGGACTTCCACAAAAATTCA.

GGTCTACTCTTATTTGCCTATCTCTATTAAACTGAAAATACAGAATAATCAAACGGATCATTCTAATAGAATTCGC-3’) via the HDOCK webserver (http://hdock.phys.hust.edu.cn/) [72].

### Electronic supplementary material

Below is the link to the electronic supplementary material.

Supplementary Material 1. Additional file 1: Fig. S1. Hydrolysis halos formed by seven *B. pumilus* strains cultivated on LB plate (+ 1% milk); Fig. S2. Comparison of hydrolytic halos between *B. subtills* wild-type and its ∆*degSU* mutant; **Fig. S3**. DNA profiles of six important plasmids in the work; **Fig. S4**. Comparison of the phenotypes of four strains of *B. pumilus*; **Fig. S5**. The 2D representations of interactions across an interface of subunits of DegU dimer (A) and DegU(L113F) dimer (B); **Fig. S6**. An overview of mutations of DegU protein reported in both literatures and this study; **Fig. S7**. Multiple sequence alignment of DegUs within the *Bacillus* lineage; **Fig. S8**. The enrichment pathway of flagellar assembly by KEGG analysis in 62 A relative to SCU11 ∆*hpr* (at 12 h); **Fig. S9**. The enrichment pathways of bacterial chemotaxis by KEGG analysis in 62 A relative to SCU11 ∆*hpr* (at 12 h).

Supplementary Material 2. Additional file 2. Table S1. The differentially expressed genes in *B. pumilus* SCU11 ∆*hpr* relative to SCU11 ∆*hpr*∆*degU* (24 h); Table S2. The differentially expressed genes in *B. pumilus* 62 A relative to SCU11 ∆*hpr*∆*degU* (24 h); **Table S3**. The differentially expressed genes clusters between DegU(WT) and DegU(L113F) (24 h); **Table S4**. The up-regulated genes in 62 A relative to ∆*hpr* (12 h); **Table S5**. The up-regulated genes in 62 A relative to ∆*hpr* (24 h); **Table S6**. The up-regulated genes in 62 A relative to ∆*hpr* (36 h); **Table S7**. The down-regulated genes in 62 A relative to ∆*hpr* (12 h); **Table S8**. The down-regulated genes in 62 A relative to ∆*hpr* (24 h); **Table S9**. The down-regulated genes in 62 A relative to ∆*hpr* (36 h); **Table S10**. The enrichment pathways of the differentially expressed genes by KEGG analysis in 62 A relative to ∆*hpr* (12 h); **Table S11**. List of major primers used in the article.

## Data Availability

The raw sequencing dataset of the *B. pumilus* SCU11 mutants is available on the NCBI Sequence Read Archive (ARS) database under accession PRJNA987134. The data supporting the conclusions of this article are included within the article and its additional files.

## Abbreviations

AprE: Major alkaline proteases, subtilisin E
DEG: Differentially expressed gene
DBD: DNA-binding domain
MD: Molecular dynamic
REC: Receiver domain
