# Supplementary material for: Tuning transcription factor DegU for developing extracellular protease overproducer in Bacillus pumilus
Source: Microb Cell Fact. 2023 Aug 27;22:163. doi: 10.1186/s12934-023-02177-0 (PMC10464342; doi:10.1186/s12934-023-02177-0)
Supplement: Supplementary file 1 — Supplementary Material 1. Additional file 1: Fig. S1. Hydrolysis halos formed by seven B. pumilus strains cultivated on LB plate (+ 1% milk); Fig. S2. Comparison of hydrolytic halos between B. subtills wild-type and its ∆degSU mutant; Fig. S3. DNA profiles of six important plasmids in the work; Fig. S4. Comparison of the phenotypes of four strains of B. pumilus; Fig. S5. The 2D representations of interactions across an interface of subunits of DegU dimer (A) and DegU(L113F) dimer (B); Fig. S6. An overview of mutations of DegU protein reported in both literatures and this study; Fig. S7. Multiple sequence alignment of DegUs within the Bacillus lineage; Fig. S8. The enrichment pathway of flagellar assembly by KEGG analysis in 62 A relative to SCU11 ∆hpr (at 12 h); Fig. S9. The enrichment pathways of bacterial chemotaxis by KEGG analysis in 62 A relative to SCU11 ∆hpr (at 12 h). [file 12934_2023_2177_MOESM1_ESM.docx]

**Supplemental materials**

**Tuning transcription factor DegU for developing extracellular protease overproducer in *B. pumilus***

Chao-Ying Xie ^1^, Wen-Jin Li ^1^, and Hong Feng^1*^

^1^Key Laboratory for Bio-resources and Eco-Environment of the Ministry of Education, Sichuan Key Laboratory of Molecular Biology and Biotechnology, College of Life Sciences, Sichuan University, Chengdu, People’s Republic of China.

*Corresponding author: hfeng@scu.edu.cn


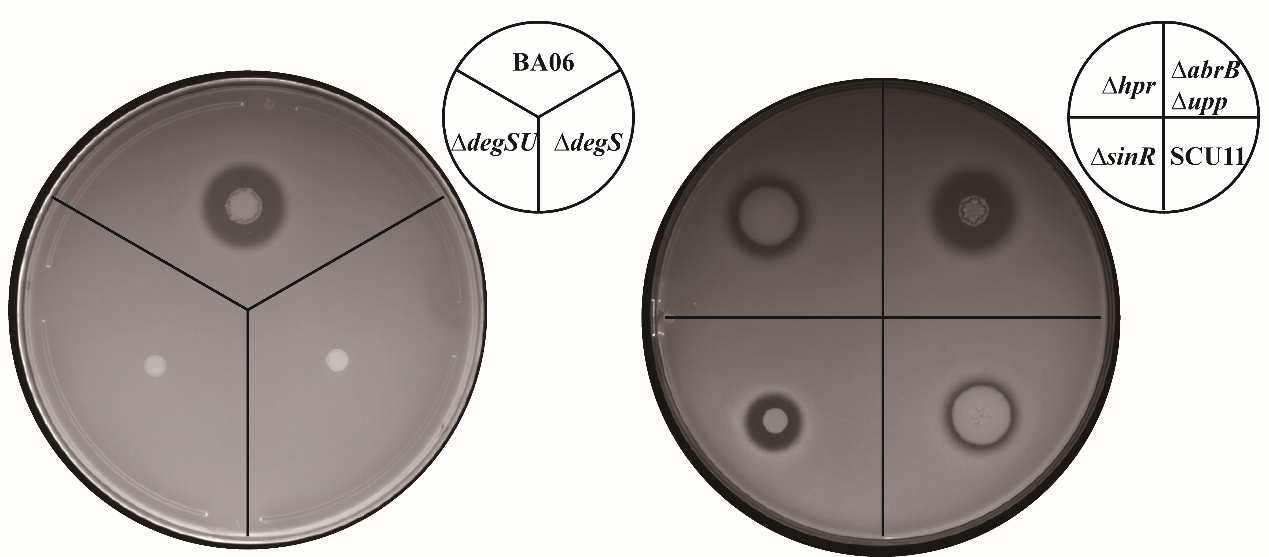


**Fig. S1**. Hydrolysis halos formed by seven *B. pumilus* strains cultivated on LB plate (+1% milk).

The overnight cultures were adjusted to the uniform density (OD600~1.0) and 1 μL droplet was inoculated on the plates (LB+1% milk). These photographs were taken after cultivated for 24 h at 37°C.


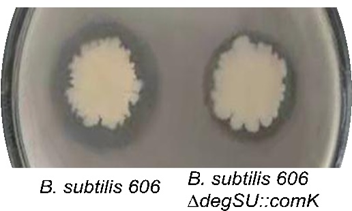


**Fig. S2**. Comparison of hydrolysis halos between *B. subtills* wild-type and its ∆*degSU* mutant.

The overnight cultures were adjusted to a uniform concentration (OD600~1.0) and 2 μL droplet was inoculated on the plates (LB+1% milk). These photographs were taken after cultivated for 24 h at 37°C.


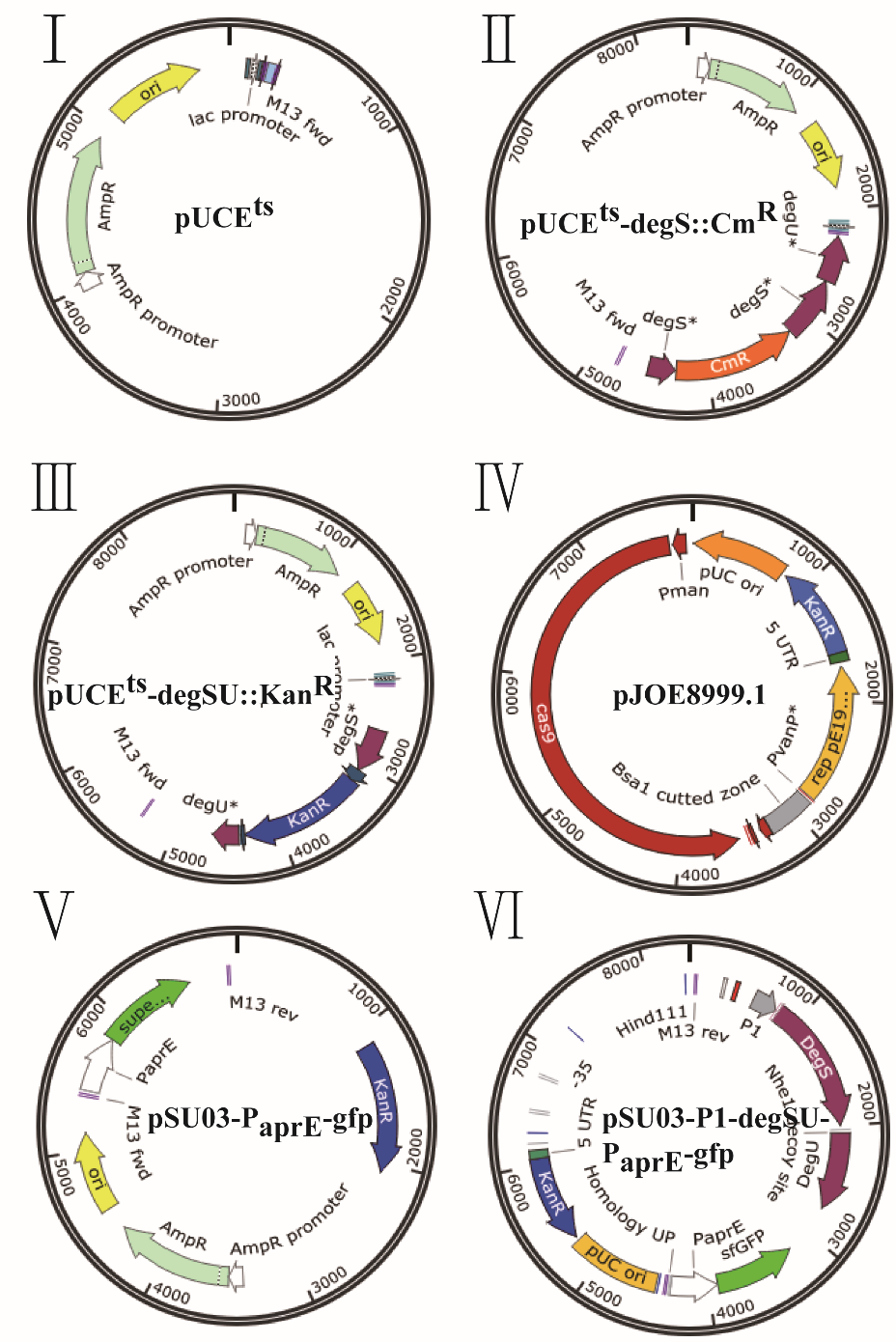


**Fig. S3**. DNA profiles of six important plasmids in the work. Briefly, for plasmid II and III, two DNA fragments (left homology arm and right homology arm) flanked its target sequences were amplified by High-fidelity DNA polymerase with primers (LH-F/LH-R, and RH-F/RH-R) using genomic DNA of *B. pumilus* SCU11 as template. The chloramphenicol resistance gene (Cm^R^) was cloned from the plasmid pHCMC02 [1]. The kanamycin resistance gene (Kan^R^) was cloned from the plasmid pSUGV4 [2]. The three fragments above were fused through overlap-PCR. The fused fragment and plasmid pUCE^ts^ were double digested by restriction endonucleases pre-selected [1]. The digested fragments were ligated using T4 DNA ligase. For plasmid V and VI, *aprE* promoter, *degSU* coding sequence were amplified by High-fidelity DNA polymerase using genomic DNA of *B. pumilus* SCU11 as template. P_1_ promoter was amplified by High-fidelity DNA polymerase using genomic DNA of *B. subtilis* 606 as template. The *gfp* reporter gene was amplified from plasmid pCN33 [3]. The P_aprE_-gfp and P_1_-degSU fragment were obtained by overlap-PCR and cloned into the plasmid pSU03-AP using homologous recombinant cloning method [4].


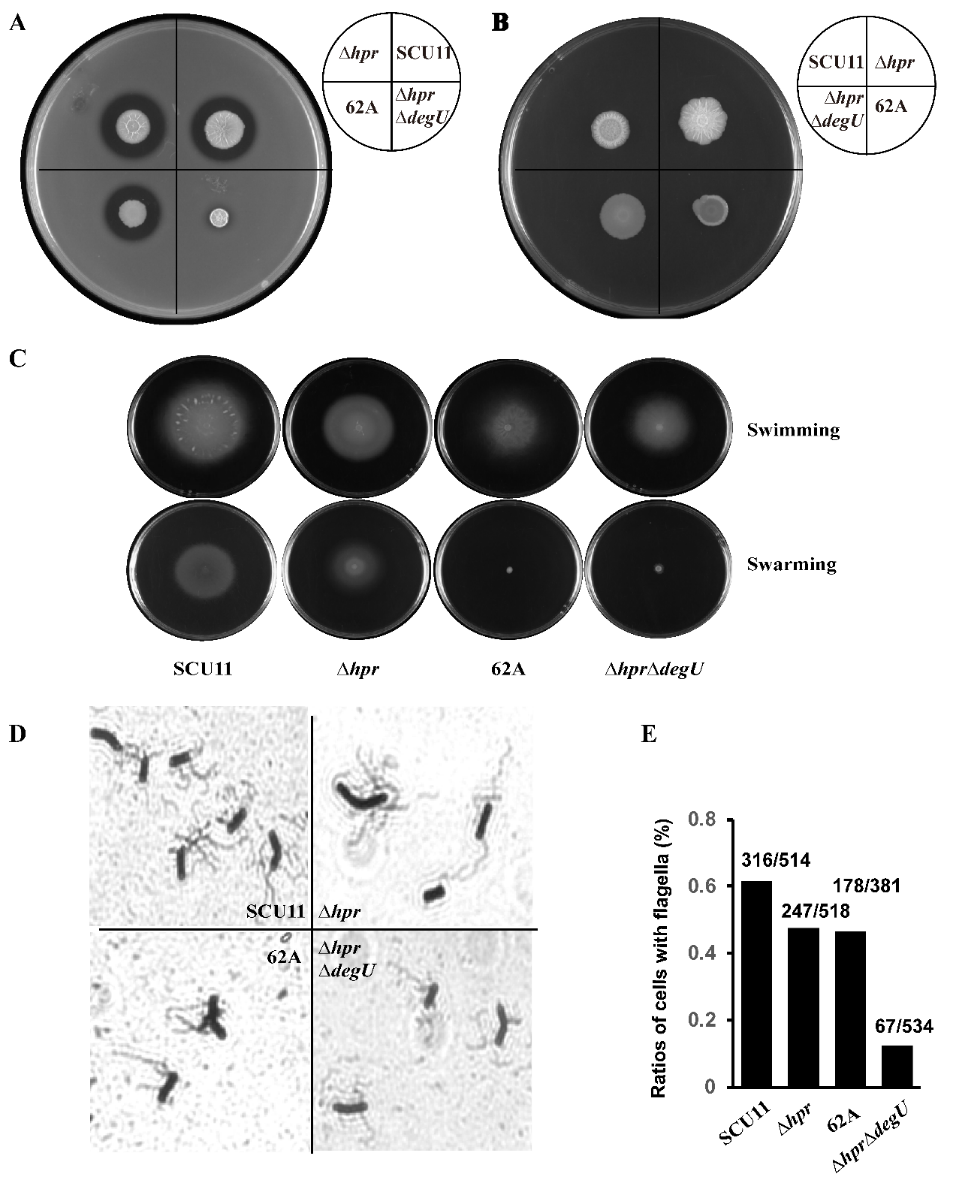


**Fig. S4**. Comparison of the phenotypes of four strains of *B. pumilus*. (A) Hydrolytic halos of SCU11, ∆*hpr*, 62A, and SCU11 ∆*hpr*/∆*degU* cultured on LB (+1% milk) plate. The overnight cultures were adjusted to the uniform density (OD600~1.0) and 1 μL droplet was inoculated on the plates for 48 h at 37°C. (B) colony morphology assay of SCU11, ∆*hpr*, 62A, and SCU11 ∆*hpr*/∆*degU* cultured on LBGM plate (LB +1% glycerol, 0.1 mM MnSO4). The overnight cultures were adjusted to the same density (OD600~1.0) and 1 μL droplet was inoculated on the plates for 48 h at 37°C. (C) Swimming and Swarming assay of SCU11, ∆*hpr*, 62A, and SCU11 ∆*hpr*/∆*degU* cultured on motility plates. The overnight cultures were adjusted to the same density (OD600~1.0) and 1 μL droplet was inoculated on the swimming plates (0.3% agar, LB based) and swarming plates (0.6% agar, LB based) for 12 h, 8 h at 37°C , respectively. The precise culture time may varied depend on the moisture of plate. (D) Flagella staining of SCU11, ∆*hpr*, 62A, and SCU11 ∆*hpr*/∆*degU*. The cultures (16 h~18 h) were properly diluted with distilled water. A drop of dilution was spread on glass slide and air-dried. The cells were stained with the solution A (5.0% tannic acid, 1.5 g% FeCl3, 0.01% NaOH, 2.0% formalin) for 5 min, subsequently with the solution B (2.0% AgNO3) for 0.5 min. After air-dried, flagella were observed at 1000 × magnification under an oil microscope. (E) Statistic of ratios of cells with flagella formation. The cells with more than 3 flagella per cell were considered as “positive” cells. The number over the bar was shown as positive cells over total cells counted.


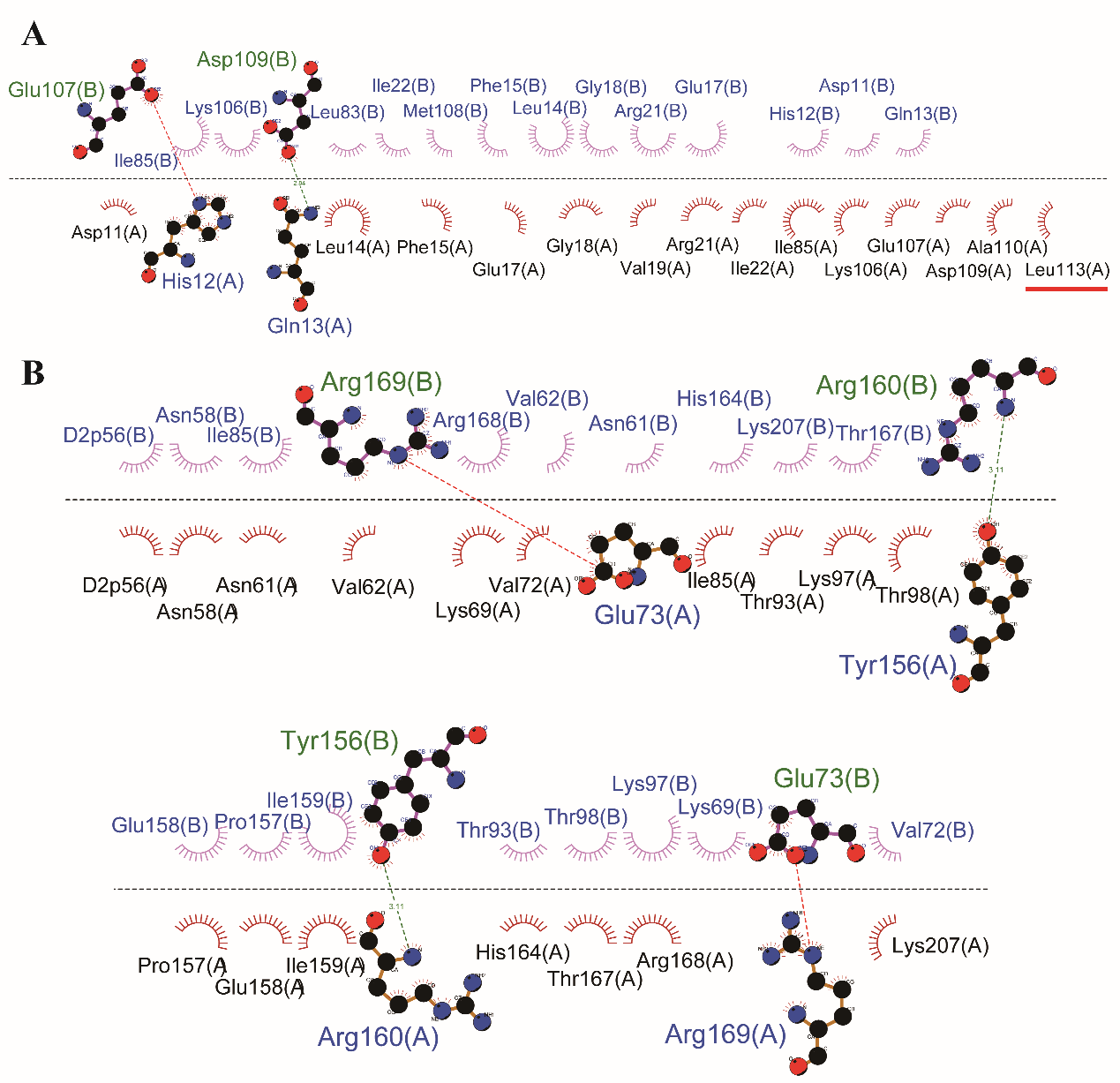


**Fig. S5**. The 2D representations of interactions across an interface of subunits of DegU dimer (A) and DegU(L113F) dimer (B). The horizontal dashed line represents the interface. The red and green dashed lines indicate the hydrogen bond. Arcs with spikes represent the residues invoving hydrophobic contacts. The letters in brackets following residues represent the chains of protein dimers. The solid dots represent atoms. The picture was plotted via software LigPlus+ v. 2.2 [5].


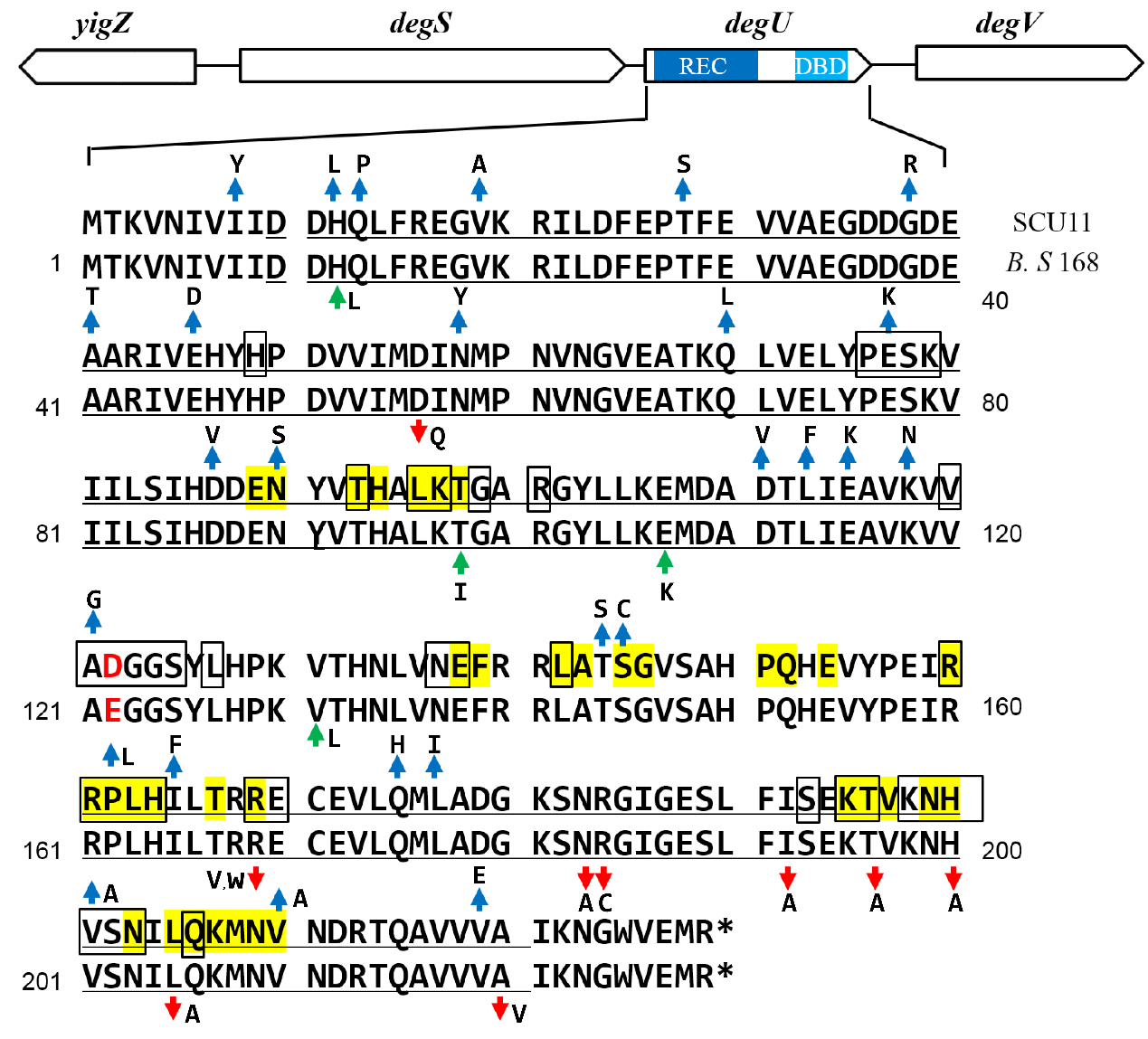


**Fig. S6**. An overview of mutations of DegU protein reported in both literatures and this study.

The DegU sequence from *B. pumilus* SCU11 and *B. subtilis* 168 were aligned together with the only difference at position 122 (D/E122). Red arrows pointing downwards represent mutations reported in prior research that abolish protease activity [6-10]. Conversely, green arrows pointing upwards represent mutations that increase protease activity [6]. The mutation sites in this study are indicated by blue arrows pointing upwards. The REC and DBD domains in DegU protein are underlined. The residues in DegU and DegU(L113F) that were predicated to interact with the *aprE* promoter are represented by the yellow highlighted and boxed letters, respectively.


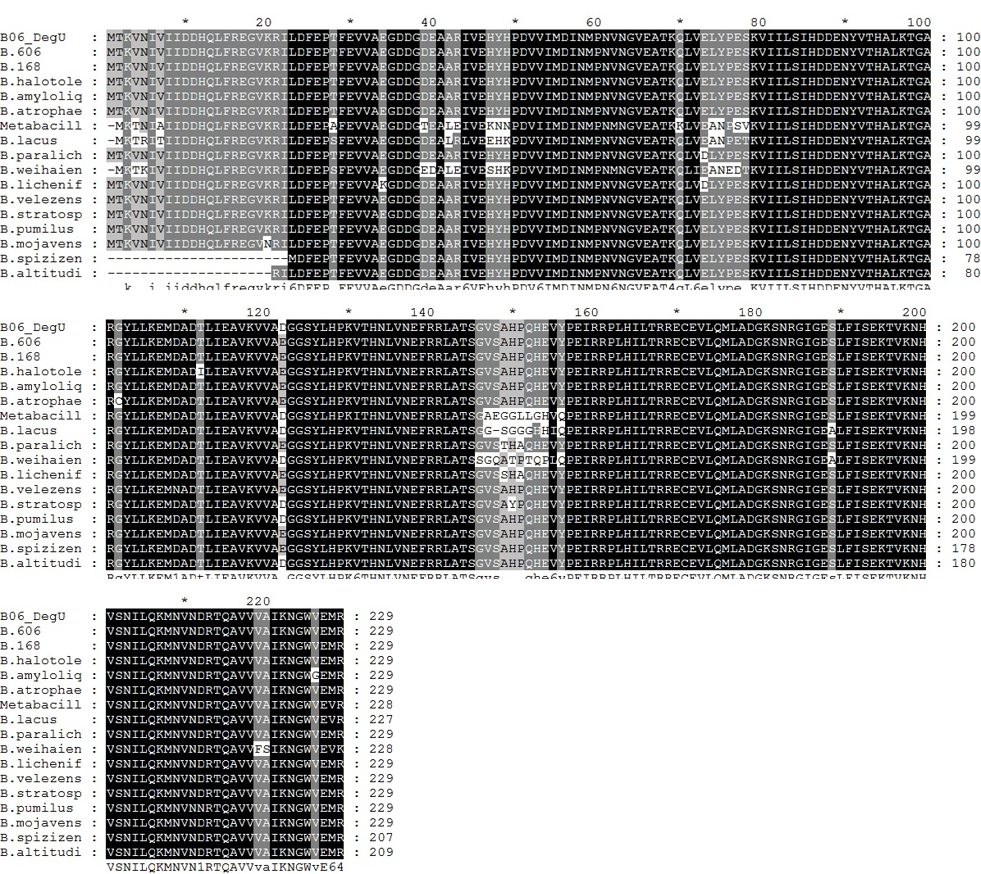


**Fig. S7**. Multiple sequence alignment of DegUs within the *Bacillus* lineage. B06_DegU, *B. pumilus* BA06 (WP_008348273.1); *B*.606, *B. subtilis* FDAARGOS_606 (WP_003219701.1); *B*.168, *B. subtilis* 168 (WP_003219701.1); *B.halotole*, *B. halotolerans* (WP_254501645.1); *B. amyloliq*, *B. amyloliquefaciens* (WP_010332071.1); *B. atrophae*, *B. atrophaeus* (WP_277780793.1); Metabacill, *Metabacillus* *sp*. GX 13764 (WP_003722647.1); *B. lacus*, *B. lacus* (WP_009331980.1); *B. paralich*, *B. paralicheniformis* (WP_165428223.1); *B.weihaien*, *B. weihaiensis* (WP_009792967.1); *B.lichienif*, *B. licheniformis* (WP_003219701.1); *B.velezens*, *B. velezensis* (WP_103042074.1); *B.stratosp*, *B. stratosphericus* (WP_226567715.1); *B.pumilus*, *B. pumilus* (WP_268453718.1); *B.mojavens*, *B. mojavensis* (WP_010332071.1); *B.spizizen*, *B. spizizenii* (WP_003219701.1); *B. altitude*, *B. altitudinis* (WP_268432169.1). MEGA 6.0 software was used to align sequences. The dark background indicated the 100% identity.


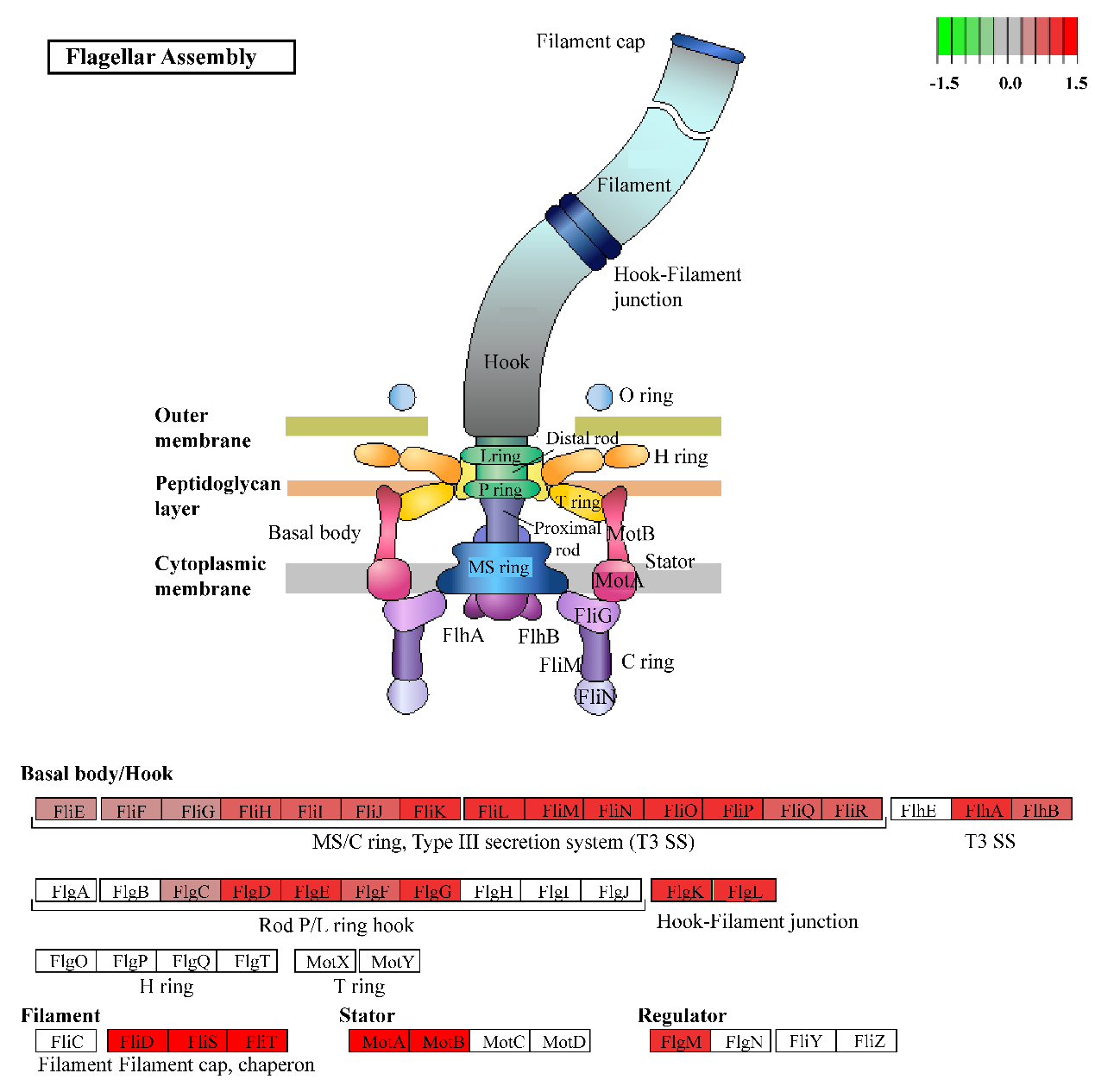


**Fig. S8**. The enrichment pathway of flagellar assembly by KEGG analysis in 62A relative to SCU11 ∆*hpr* (at 12 h). The heatmap of KEGG pathways according to the.log_2_FoldChange values of Fisher’s exact test was obtained from enrichment analysis. The picture was generated through the pathview package. More enrichment information can be acquired in additional file 2: **Table S10**.


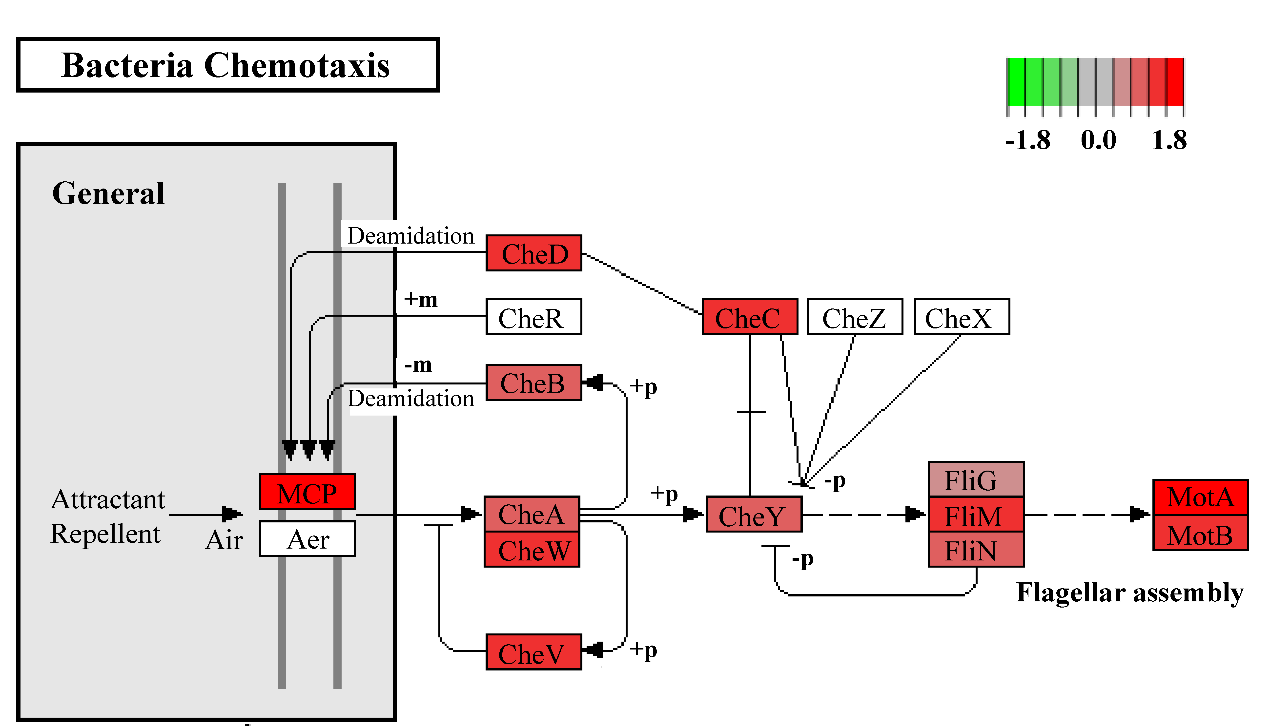


**Fig. S9**. The enrichment pathways of bacterial chemotaxis by KEGG analysis in 62A relative to SCU11 ∆*hpr* (at 12 h). The heatmap of KEGG pathways according to the.log_2_FoldChange values of Fisher’s exact test was obtained from enrichment analysis. In this graph, +p (phosphorylation reaction); -p (dephosphorylation reaction); +m (methylation reaction); -m (demethylation); MCP (methyl-accepting chemotaxis protein). Lines with arrow heads indicate activation, while lines with bar heads indicate inhibition. The picture was generated through the pathview package. More enrichment information can be acquired in additional file-Table S10.

References

1. Han LL, Liu YC, Miao CC, Feng H. Disruption of the pleiotropic gene *scoC* causes transcriptomic and phenotypical changes in *Bacillus pumilus* BA06. BMC Genom. 2019; 20:327.

2. He MX, Feng H, Zhang YZ. Construction of a novel cell-surface display system for heterologous gene expression in *Escherichia coli* by using an outer membrane protein of *Zymomonas mobilis* as anchor motif. Biotechnol Lett. 2008; 30:2111-2117.

3. Ivain L, Bordeau V, Eyraud A, Hallier M, Dreano S, Tattevin P, Felden B, Chabelskaya S. An in vivo reporter assay for sRNA-directed gene control in Gram-positive bacteria: identifying a novel sRNA target in *Staphylococcus aureus*. Nucleic Acids Res. 2017; 45:4994-5007.

4. Shao H, Cao Q, Zhao H, Tan X, Feng H. Construction of novel shuttle expression vectors for gene expression in *Bacillus subtilis* and *Bacillus pumilus*. J Gen Appl Microbiol. 2015; 61:124-131.

5. Laskowski RA, Swindells MB. LigPlot^+^: multiple ligand-protein interaction diagrams for drug discovery. J Chem Inf Model. 2011; 51:2778-2786.

6. Msadek T, KUNST F, Henner D, Klier A, Rapoport G, Dedonder R. Signal transduction pathway controlling synthesis of a class of degradative enzymes in *Bacillus subtilis*: expression of the regulatory genes and analysis of mutations in *degS* and *degU*. J Bacteriol. 1990; 172:824-834.

7. Shimane K, Ogura M. Mutational analysis of the helix-turn-helix region of *Bacillus subtilis* response regulator DegU, and identification of *cis*-acting sequences for DegU in the *aprE* and *comK* promoters. J Biol Chem. 2004; 136:387-397.

8. Kobayashi K. Gradual activation of the response regulator DegU controls serial expression of genes for flagellum formation and biofilm formation in *Bacillus subtilis*. Mol Microbiol. 2007; 66:395-409.

9. Trott AE, Stevens AM. Amino acid residues in LuxR critical for its mechanism of transcriptional activation during quorum sensing in *Vibrio fischeri*. J Bacterial. 2001; 183:387-392.

10. Gupta M, Rao KK. Phosphorylation of DegU is essential for activation of *amyE* expression in *Bacillus subtilis*. J Biosci. 2014; 39:747-752.
